# Reply to Charles V. Vorhees: Aspartame and anxiety-like behavior

**DOI:** 10.1073/pnas.2304679120

**Published:** 2023-06-05

**Authors:** Sara K. Jones, Deirdre M. McCarthy, Cynthia Vied, Gregg D. Stanwood, Chris Schatschneider, Pradeep G. Bhide

**Affiliations:** ^a^Biomedical Sciences, Florida State University, Tallahassee, FL 32306; ^b^Translational Science Laboratory, Florida State University, Tallahassee, FL 32306; ^c^Psychology, Florida State University, Tallahassee, FL 32306

Jones et al. (1) exposed male and female mice to three aspartame concentrations and examined anxiety-like behavior using the open field test (OFT) and elevated zero maze (EZM). The study design was asymmetrical to afford statistical power to key analyses of transgenerational transmission along paternal lineage. Thus, not all mice were examined in both OFT and EZM; F0 males (but not females) exposed to a subset of aspartame concentrations produced the F1 and F2 generations; and only male mice were examined from the lowest aspartame concentration. A sensitivity analysis of the litter effect produced intraclass correlation coefficient at or near zero, demonstrating lack of effect. Nevertheless, in response to the Letter to the Editor by Vorhees (2), analyses incorporating litter, sex, and aspartame dose are reported below, which corroborate the published findings (1).

The center time in OFT, analyzed with litter as a random factor using a 3 × 2 mixed-model ANOVA for F1 and a 2 × 2 mixed-model ANOVA for F2 (only 0.03% lineage in F2), showed a significant effect of lineage [F1: F_(2,39)_ = 160.82; *P* < 0.0001; F2: F_(1,22)_ = 6.63, *P* < 0.05] and no significant effect of sex or sex × lineage interaction.

For F0, a 3 × 2 × 5 mixed-model ANOVA confirmed significant effects of drinking water treatment [F_(2,210)_ = 117.41, *P* < 0.0001] and drinking water × testing interval interaction [F_(8,210)_ = 14.27, *P* < 0.0001] for the center time in OFT. Post hoc comparisons demonstrated significant effects of drinking water treatment beginning at 6 wk, confirming the published findings (1). When two doses of diazepam were analyzed in a 3 × 2 × 2 ANOVA, with litter as a random factor for F1, there was a significant effect of diazepam [F0: F_(1,76)_= 25.85, *P* < 0.0001; F1: F_(1,75)_ = 19.82, *P* < 0.0001] and no significant effect of sex or sex × drug interaction.

Jones et al. (1) demonstrated transgenerational transmission but did not claim evidence of mechanisms; epigenetic changes in spermatozoa were discussed as potential candidates. RNA sequencing was performed only in F0 males (founders of the paternal lineages) at one aspartame dose, but quantitative PCR was performed in male and female F0, F1, and F2 mice and was appropriately powered (1).

Vorhees cites a meta-analysis (3) of studies using benzodiazepines (5 compounds), antidepressants (12 compounds), and “other mechanisms” (1 compound). Since antidepressants rather than anxiolytics were the majority of drugs analyzed, the poor predictive validity suggested by the analysis is not surprising. In fact, rodent models of anxiety-related behavior were developed deliberately to differentiate anxiolytics from antidepressants. Moreover, many studies including Vorhees (4–7) used OFT and EZM to analyze anxiety-like behavior. Aspartame-induced anxiety-like behavior was described in rats previously (8), offering the “validation” in a “second species” sought in the Letter to the Editor by Vorhees (2).

Finally, Jones et al. (1) stated that aspartame per se is unlikely to induce anxiety-like behavior because little intact aspartame likely entered the brain and discussed the lack of consensus in the literature on the mechanisms underlying aspartame’s behavioral effects.

The additional analyses presented here further demonstrate the robustness of the published findings (1). Replication and extension of these findings may further corroborate the potential deleterious effects of aspartame consumption.
